# The Association Between Admission Sources and Outcomes at a Pediatric Intensive Care Unit in Al-Ahsa, Saudi Arabia: A Retrospective Cohort Study

**DOI:** 10.7759/cureus.11356

**Published:** 2020-11-05

**Authors:** Sajjad M AlKadhem, Sami AlKhwaitm, Ahmed Z Alkhars, Nasir Al Dandan, Wejdan Almarzooq, Hassan Al Bohassan, Fatimah A AlMuhanna

**Affiliations:** 1 Pediatrics, Maternity and Children Hospital Al-Ahsa, Al-Ahsa, SAU; 2 Pediatric Critical Care Medicine, Maternity and Children Hospital Al-Ahsa, Al-Ahsa, SAU; 3 Pediatrics, College of Medicine, King Faisal University, Al-Ahsa, SAU; 4 Medicine, College of Medicine, King Faisal University, Al-Ahsa, SAU; 5 Pediatrics, King Faisal University, Al-Ahsa, SAU; 6 Medicine, King Faisal University, Al-Ahsa, SAU

**Keywords:** epidemiology, case-specific mortality, pediatric icu, saudi arabia, source of admission

## Abstract

Objectives

In this study, we aimed to examine the association between sources of admission (either intra-hospital transfers or ED admissions) in pediatric intensive care units (PICUs) and the discharge rate, mortality rate, and referral over a period of three years. We also sought to identify the independent predictors of discharge and mortality rate in the study population.

Patients and methods

This was a retrospective cohort study involving the analysis of 2,547 patients' data collected from the Pediatric Intensive Care Registry of a secondary care community hospital. We included patients admitted to the PICU from January 1, 2016, till December 31, 2018, who were aged 0-14 years with a specific diagnosis, recorded source of admission, and clear outcome. Data were collected, coded, and analyzed using the SPSS Statistics software (IBM, Armonk, NY) and STATA software (StataCorp, College Station, TX).

Results

Of the included patients, 1,356 (53.2%) were males, and 1,191 (46.8%) were females. Infants were associated with an increased risk of a long stay in the hospital [relative risk ratio (RRR)=5.34, 95% CI: (1.28, 22.27)] and mortality [RRR=3.56, 95% CI: (1.41, 8.95)] compared to older children. Similarly, neonates were associated with a higher risk of mortality [RRR=2.83, 95% CI: (1.05, 7.65)]. Patients who were admitted through ED were associated with a lower risk of a long-stay [RRR=0.56, 95% CI: (10.36, 0.87)] and mortality [RRR=0.68, 95% CI: (0.49, 0.95)] compared to intra-hospital transfers. Concerning the admission date, all time periods were associated with a lower risk of mortality compared to the period of October-December.

Conclusion

Our findings showed that the age of patients, source of admission, and date of admission might be used as independent predictors for determining the outcome of admissions, including discharge and mortality rates. Further studies are required to confirm these findings.

## Introduction

Fortunately, few children need intensive care at the hospital; however, those who do are likely to suffer a high risk of morbidity and mortality [1,2]. In pediatric critical care, childhood morbidity and mortality are particularly challenging problems, which require effective coordination between burdensome and invasive interventions to support recovery and avoid un-survivable illnesses [3-5]. Recently, medical advances in treating multiple developmental conditions have contributed to lower deaths. Consequently, more children survive with chronic comorbidities. Such clinical advances demand an understanding of the evolving mortality trends and call for a wider review of the related social, legal, and economic issues, along with the goals and values of families and clinicians [6,7].

Studies conducted in different countries have indicated the association between the sources of admissions in pediatric intensive care units (PICUs) with morbidity and mortality rates [3,7-10]. The pediatric admission sources may be intra-hospital transfers or admissions through EDs [11]. It was reported that patients transferred from hospital wards were associated with greater ICU mortality than those transferred from other sources [12,13]. Moreover, some studies have demonstrated that pediatric patients transferred from other hospitals to PICUs are generally associated with worse conditions compared to those transferred from the same hospital [14-16]. With the recent focus on hospital mergers and the consolidation of clinical facilities within tertiary centers, the transportation of patients between hospitals will continue to be an existing feature of healthcare delivery systems, and it will become increasingly necessary to assess whether variations in access to intensive care have an impact on patient outcomes. Therefore, this study aimed to explore the association of admission sources, age, and diagnosis with clinical outcomes in PICUs.

## Materials and methods

Study design and setting

This was a retrospective cohort study involving the analysis of 2,547 patients' data collected from the Pediatric Intensive Care Registry of a secondary care community hospital [Maternity and Children Hospital, Al-Ahsa (MCH-Al-Ahsa), Saudi Arabia]. The MCH-Al-Ahsa is a 450-bed hospital specializing in obstetrics and gynecology, neonatology, and pediatrics. The PICU of the hospital is a 20-bed unit with an average occupancy rate of 80-85%. Given the retrospective nature of the study, written informed consent was not considered. The patient-related data were collected in an anonymized database, and during analysis, the patients' privacy was ensured.

Inclusion and exclusion criteria

Patients admitted to the PICU from January 1, 2016, till December 31, 2018, who were aged 0-14 years with a specific diagnosis, recorded sources of admission, and clear outcomes were included in this study. Patients with incomplete data (no specific diagnosis, source of admission not mentioned, outcomes not mentioned) were excluded; patients admitted for social, non-medical reasons were also excluded.

Data collection

The children were classified into different groups according to their age, health analysis, and outcomes. The age groups were classified as follows: neonates (zero days to one month), infants (one month to one year), toddlers (one to three years), pre-school children (three to six years), school-age children (referred to as school-age; 6-12 years) and adolescents (referred to as older children; 12-14 years). We recorded the following items for analysis: age, gender, admission sources, duration of admission, discharge rate, and the reported mortality rate.

Statistical analysis

Data were coded and analyzed using the SPSS Statistics software, version 24 for Windows (IBM, Armonk, NY). Categorical data were presented as frequencies and percentages. A chi-square test was performed to evaluate the difference between studied categories. The STATA software version 15 (StataCorp, College Station, TX) was used for conducting the multinomial logistic regression. A p-value of less than 0.05 was considered statistically significant.

## Results

Demographic characteristics 

Of the included patients, 1,356 (53.2%) were males, and 1,191 (46.8%) were females. The majority of included patients were Saudi nationals (2,457, 96.5%). Approximately, 12% were neonates, 33.4% infants, 20.1% toddlers, 8.2% pre-school, 18.7% school-age, and 7.6% older children. Table 1 shows the demographic characteristics of the included patients.

**Table 1:** Demographic characteristics of included patients

Demographic characteristics		N	%
Gender	Male	1,356	53.2%
	Female	1,191	46.8%
Nationality	Saudi	2,457	96.5%
	Non-Saudi	90	3.5%
Age group	Infant	850	33.4%
	Neonate	305	12.0%
	Toddler	513	20.1%
	Pre-school	210	8.2%
	School-age	476	18.7%
	Older	193	7.6%

Association between admissions and demographic characteristics 

**Figure 1:**
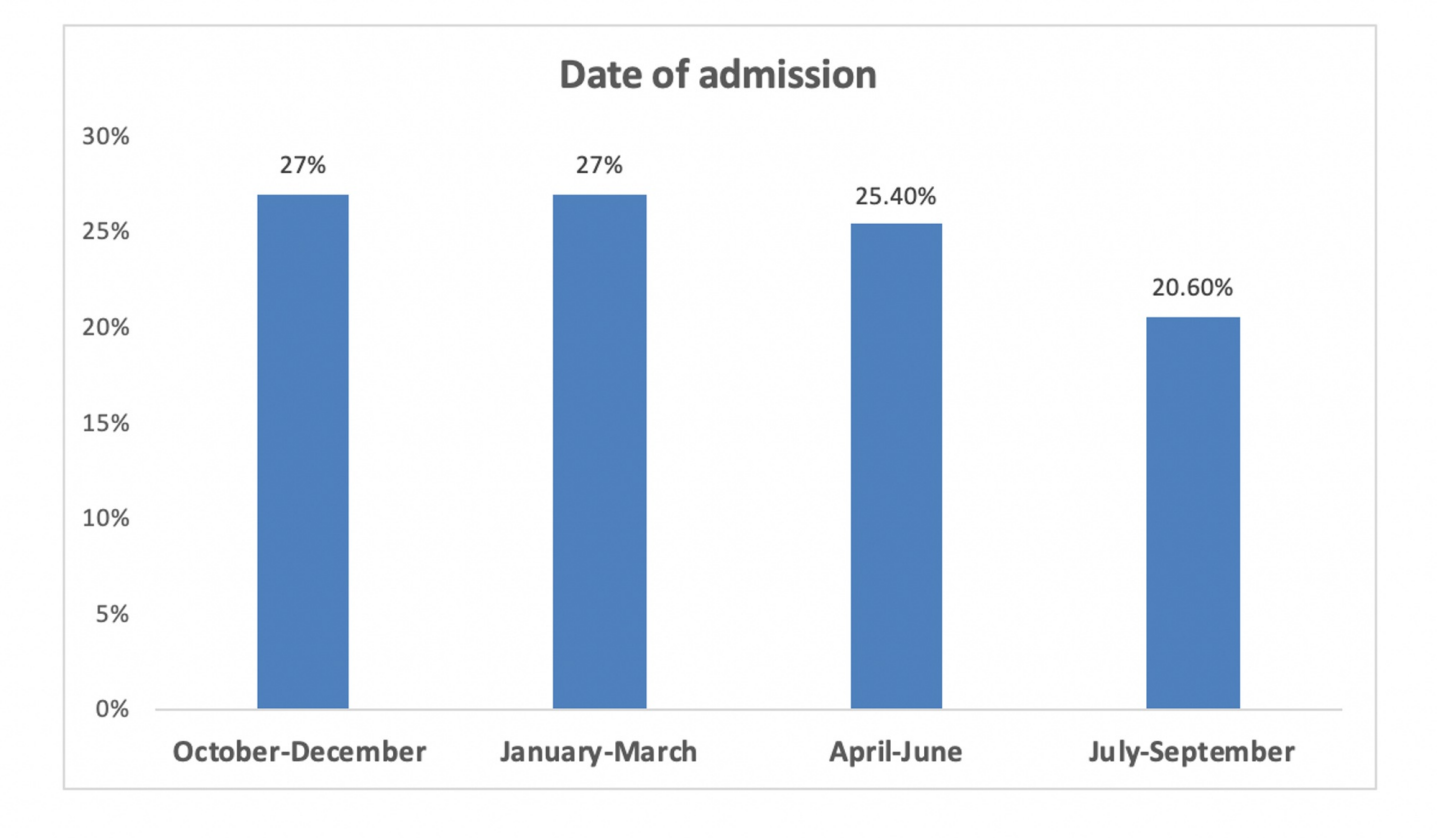
Admission dates

In terms of the source of admission, 1,552 (60.9%) of the included patients were admitted via the ED, and 995 (39.1%) were admitted through intra-hospital transfers. The rate of admission was constantly the highest during October-March (27%). It showed a slight reduction in April-June (25.4%) and July-September (20.6%). Figure 1 shows the data related to admission dates.

Our analysis showed no significant difference between both genders in terms of the sources of admission (p=0.625). Moreover, the percentage rates of admission between Saudi and non-Saudi patients were comparable (p=0.124). On the other hand, we found a significant difference (p<0.001) between both admission sources according to the age class; for instance, the infant admission rate was higher for intra-hospital transfers (Table 2). Regarding the admission date, the ED admission recorded a higher admission rate on all dates; however, the difference was not significant (p=0.224). Concerning the association between demographic characteristics and admission dates, we could not find any significant association with gender (p=0.573) and nationality (p=0.083), while the patients' age was significantly associated with the admission date; for infants and neonates, a higher rate was observed in January-March. The same was observed in April-June for pre-school children, in July-September for school-age, and October-December for toddlers and older children (Table 3).

**Table 2:** Association between admission source and demographic data

Parameters		Emergency department	Intra-hospital transfer	P-value
Gender	Male	820 (60.5%)	536 (39.5%)	0.625
	Female	732 (61.5%)	459 (38.5%)	
Nationality	Saudi	1,490 (60.6%)	967 (39.4%)	0.124
	Non-Saudi	62 (68.9%)	28 (31.3%)	
Age group	Infant	424 (49.9%)	426 (50.1%)	<0.001
	Neonate	204 (66.9%)	101 (33.1%)	
	Toddler	332 (64.7%)	181 (35.3%)	
	Pre-school	135 (64.3%)	75 (35.7%)	
	School-age	318 (66.8%)	158 (33.2%)	
	Older	139 (72%)	54 (28%)	
Admission date	January-March	399 (58%)	289 (42%)	0.224
	April-June	401 (62%)	246 (38%)	
	July-September	333 (63.4%)	192 (36.6%)	
	October-December	419 (61%)	268 (39%)	

**Table 3:** Association between admission date and demographic data

Parameters		January-March	April-June	July-September	October-December	P-value
Gender	Male	355 (26.2%)	346 (25.5%)	292 (21.5%)	363 (26.8%)	0.573
	Female	333 (28%)	301 (25.3%)	233 (19.6%)	324 (27.2%)	
Nationality	Saudi	659 (26.8%)	617 (25.1%)	511 (20.8%)	670 (27.3%)	0.083
	Non-Saudi	29 (32.2%)	30 (33.3%)	14 (15.6%)	17 (18.9%)	
Age group	Infant	253 (29.8%)	201 (23.6%)	156 (18.4%)	240 (28.2%)	<0.001
	Neonate	104 (34.1%)	79 (25.9%)	57 (18.7%)	65 (21.3%)	
	Toddler	142 (27.7%)	132 (25.7%)	96 (18.7%)	143 (27.9%)	
	Pre-school	41 (19.5%)	65 (31.0%)	50 (23.8%)	54 (25.7%)	
	School-age	113 (23.7%)	111 (23.3%)	127 (26.7%)	125 (26.3%)	
	Older	35 (18.1%)	59 (30.6%)	39 (20.2%)	60 (31.1%)	

Diagnosis of included patients during admission

Regarding the clinical condition, we used the online version of the World Health Organization (WHO) International Classification of Diseases, Tenth Revision (ICD-10) to classify our patients. Respiratory diseases were the most frequently encountered condition (21.3%) among the population in this study, followed by endocrine, nutritional, and metabolic diseases (16.7%), certain infectious and parasitic diseases (12.2%), diseases of the nervous system (10.2%), diseases of the circulatory system, diseases of the blood and blood-forming organs and certain disorders involving the immune mechanism (7.6%), and injury, poisoning, and certain other consequences of external causes (7%). Table 4 summarizes the clinical conditions of the included patients.

**Table 4:** Diagnosis of included patients during admission according to the online version of WHO ICD-10 (2019) WHO ICD-10: World Health Organization International Classification of Diseases, Tenth Revision

WHO ICD-10 classification	Frequency	Percent
ICD-10 (I): Certain infectious and parasitic diseases	311	12.2%
ICD-10 (II): Neoplasms	6	0.2%
ICD-10 (III): Diseases of the blood and blood-forming organs and certain disorders involving the immune mechanism	193	7.6%
ICD-10 (IV): Endocrine, nutritional, and metabolic diseases	426	16.7%
ICD-10 (VI): Diseases of the nervous system	260	10.2%
ICD-10 (VIII): Diseases of the ear and mastoid process	1	0.0003%
ICD-10 (IX): Diseases of the circulatory system	254	10.0%
ICD-10 (X): Diseases of the respiratory system	542	21.3%
ICD-10 (XI): Diseases of the digestive system	80	3.1%
ICD-10 (XII): Diseases of the skin and subcutaneous tissue	4	0.2%
ICD-10 (XIII): Diseases of the musculoskeletal system and connective tissue	72	2.82%
ICD-10 (XIV): Diseases of the genitourinary system	36	1.4%
ICD-10 (XVI): Certain conditions originating in the perinatal period	101	4.0%
ICD-10 (XVII): Congenital malformations, deformations, and chromosomal abnormalities	17	0.7%
ICD-10 (XVIII): Symptoms, signs, and abnormal clinical and laboratory findings, not classified elsewhere	41	1.6%
ICD-10 (XIX): Injury, poisoning, and certain other consequences of external causes	178	7.0%
ICD-10 (XX): External causes of morbidity and mortality	21	0.8%
ICD-10 (XXI): Factors influencing health status and contact with health services	4	0.2%
Total	2,547	100%

Outcomes of admission

**Figure 2:**
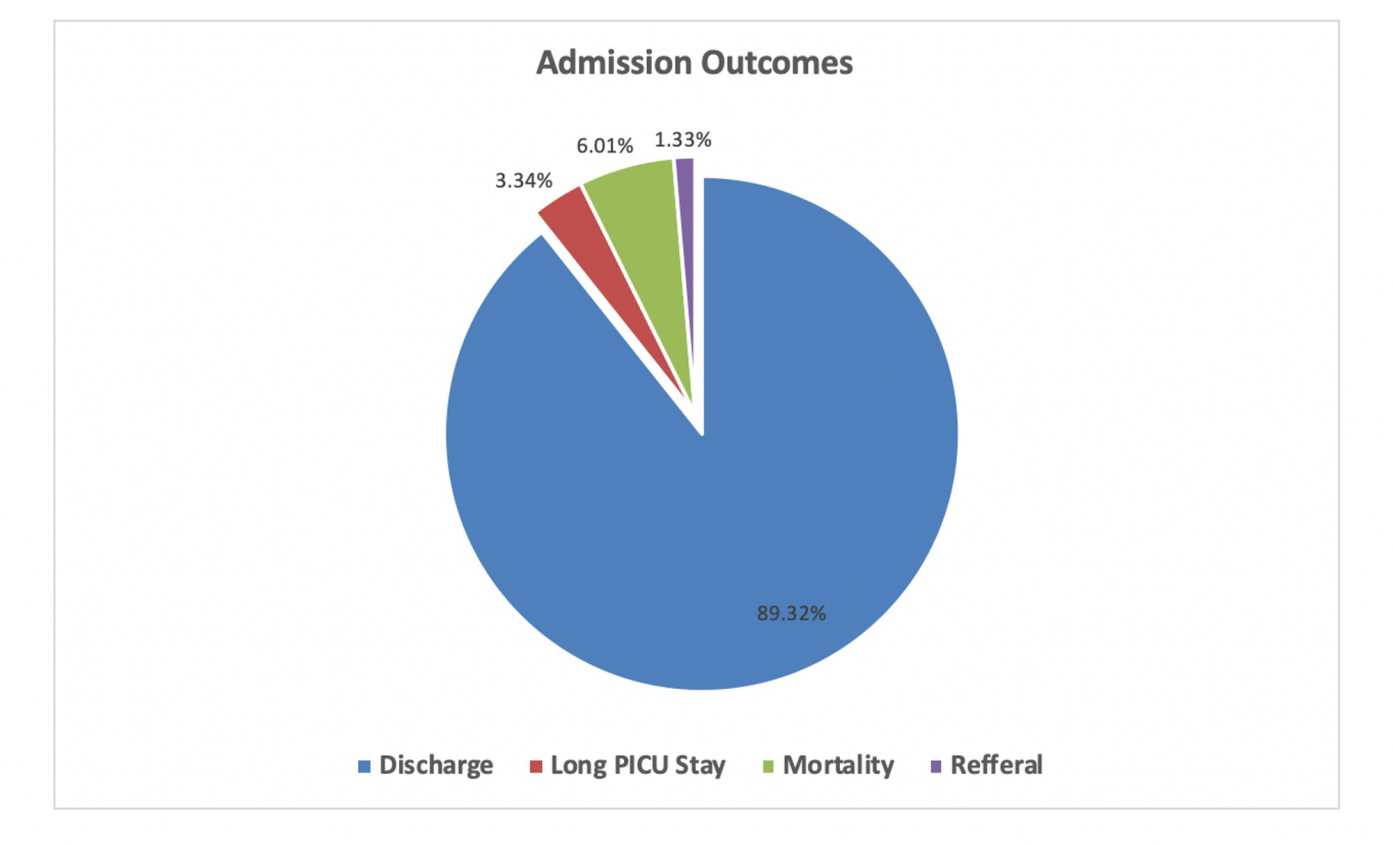
Admission outcomes PICU: pediatric intensive care unit

Fortunately, 89.32% of the admitted patients were discharged home; however, 6.01% of the patients died, 3.34% had long PICU stay, and 1.33% were referred to another hospital (Figure 2). Our analysis demonstrated no significant differences in the gender (p=0.458) and the nationality (p=0.447) of the included patients concerning the outcomes. On the other hand, infants and neonates were associated with a higher mortality rate (8.2% and 6.9%, respectively). Moreover, school-age and older children were associated with the highest discharge rate and a lower mortality rate (p=0.001). Similarly, patients who were transferred from the ED were associated with a lower mortality rate (5.2% vs. 7.2%) and duration of stay (2.6% vs. 4.4%) compared to intra-hospital transfers (p=0.007). In terms of admission date, the highest mortality rate was observed in those admitted during the period of October-December (9.8%), whereas the highest rate of discharge was observed in those admitted during the period of April-June (91%). Regarding the diagnosis, the highest cause-specific mortality rates (but not the total number) were associated with congenital malformations, deformations, and chromosomal abnormalities (23.5%), followed by neoplasms (16.7%), external causes of morbidity and mortality (14.3%), circulatory disease (13%), and infectious disease (12.5%). Table 5 summarizes the factors associated with admission outcomes. 

**Table 5:** Association of admission date and demographic data with admission outcomes PICU: pediatric intensive care unit; ED: emergency department; ICD-10: International Classification of Diseases, Tenth Revision

Parameters		Discharge, n (%)	Long PICU stay, n (%)	Mortality, n (%)	Referral, n (%)	P-value
Gender	Male	1,221 (90%)	39 (2.9%)	80 (5.9%)	16 (1.2%)	0.458
	Female	1,054 (88.5%)	46 (3.9%)	73 (6.1%)	18 (1.5%)	
Nationality	Saudi	2,198 (89.5%)	82 (3.3%)	144 (5.9%)	33 (1.3%)	0.447
	Non-Saudi	77 (85.6%)	3 (3.3%)	9 (10.0%)	1 (1.1%)	
Age group	Infant	727 (85.5%)	42 (4.9%)	70 (8.2%)	11 (1.3%)	0.001
	Neonate	274 (89.8%)	7 (2.3%)	21 (6.9%)	3 (1%)	
	Toddler	462 (90.1%)	14 (2.7%)	28 (5.5%)	9 (1.8%)	
	Pre-school	183 (87.1%)	11 (5.2%)	12 (5.7%)	4 (1.9%)	
	School-age	444 (93.3%)	9 (1.9%)	17 (3.6%)	6 (1.3%)	
	Older	185 (95.9%)	2 (1%)	5 (2.6%)	1 (0.5%)	
Source of admission	ED admission	1412 (91%)	41 (2.6%)	81 (5.2%)	18 (1.2%)	0.007
	Intra-hospital	863 (86.7%)	44 (4.4%)	72 (7.2%)	16 (1.6%)	
Date of admission	January-March	619 (90%)	27 (3.9%)	32 (4.7%)	10 (1.5%)	0.002
	April-June	589 (91%)	24 (3.7%)	25 (3.9%)	9 (1.4%)	
	July-September	474 (90.3%)	15 (2.9%)	29 (5.5%)	7 (1.3%)	
	October-December	593 (86.3%)	19 (2.8%)	67 (9.8%)	8 (1.2%)	
Diagnosis	ICD-10 (I): Certain infectious and parasitic diseases	259 (83.3%)	10 (3.2%)	39 (12.5%)	3 (1%)	<0.001
	ICD-10 (II): Neoplasms	3 (50%)	1 (16.7%)	1 (16.7%)	1 (16.7%)	
	ICD-10 (III): Diseases of the blood and blood-forming organs and certain disorders involving the immune mechanism	179 (92.7%)	4 (2.1%)	6 (3.1%)	4 (2.1%)	
	ICD-10 (IV): Endocrine, nutritional, and metabolic diseases	416 (97.7%)	2 (0.5%)	8 (1.9%)	0 (0%)	
	ICD-10 (VI): Diseases of the nervous system	219 (84.2%)	19 (7.3%)	18 (6.9%)	4 (1.5%)	
	ICD-10 (VIII): Diseases of the ear and mastoid process	1 (100%)	0 (0%)	0 (0%)	0 (0%)	
	ICD-10 (IX): Diseases of the circulatory system	206 (81.1%)	12 (4.7%)	33 (13%)	3 (1.2%)	
	ICD-10 (X): Diseases of the respiratory system	501 (92.4%)	22 (4.1%)	15 (2.8%)	4 (0.7%)	
	ICD-10 (XI): Diseases of the digestive system	69 (86.3%)	1 (1.3%)	4 (5%)	6 (7.5%)	
	ICD-10 (XII): Diseases of the skin and subcutaneous tissue	3 (75%)	1 (25%)	0 (0%)	0 (0%)	
	ICD-10 (XIII): Diseases of the musculoskeletal system and connective tissue	72 (100%)	0 (0%)	0 (0%)	0 (0%)	
	ICD-10 (XIV): Diseases of the genitourinary system	30 (83.3%)	2 (5.6%)	1 (2.8%)	3 (8.3%)	
	ICD-10 (XVI): Certain conditions originating in the perinatal period	99 (98%)	1 (1%)	1 (1%)	0 (0%)	
	ICD-10 (XVII): Congenital malformations, deformations, and chromosomal abnormalities	12 (70.6%)	1 (5.9%)	4 (23.5%)	0 (0%)	
	ICD-10 (XVIII): Symptoms, signs, and abnormal clinical and laboratory findings, not classified elsewhere	37 (90.2%)	0 (0%)	3 (7.3%)	1 (2.4%)	
	ICD-10 (XIX): Injury, poisoning, and certain other consequences of external causes	149 (83.7%)	8 (4.5%)	16 (9%)	5 (2.8%)	
	ICD-10 (XX): External causes of morbidity and mortality	17 (81%)	1 (4.8%)	3 (14.3%)	0 (0%)	
	ICD-10 (XXI): Factors influencing health status and contact with health services	4 (100%)	0 (0%)	0 (0%)	0 (0%)	

Multinomial logistic regression 

Both gender and nationality showed non-significant regression. Regarding the age groups, infants were associated with increased risk of long-stay [relative risk ratio (RRR)=5.34, 95% CI: (1.28, 22.27)] and mortality [RRR=3.56, 95% CI: (1.41, 8.95)] compared to other age groups. Similarly, neonates were also associated with a higher risk of mortality [RRR=2.83, 95% CI: (1.05, 7.65)].

In contrast, patients who were admitted through ED were associated with a lower risk of long-stay [RRR=0.56, 95%: CI (10.36, 0.87)] and mortality [RRR=0.68, 95% CI: (0.49, 0.95)] compared to intra-hospital transfers. Concerning the admission date, all time periods were associated with a lower risk of mortality compared to October-December. Table 6 shows the results of multinomial logistic regression.

**Table 6:** Multinomial logistic regression RRR: relative risk ratio; ED: emergency department; ICD-10: International Classification of Diseases, Tenth Revision

Parameters		Long-stay, RRR (95% CI)	Mortality, RRR (95% CI)	Referral, RRR (95% CI)
Gender	Male	Ref		
	Female	1.36 (0.88, 2.10)	1.05 (0.76, 1.46)	1.30 (0.66, 2.56)
Nationality	Saudi	0.95 (0.29, 3.09)	0.56 (0.27, 1.14)	1.15 (0.15, 8.56)
	Non-Saudi	Ref		
Age group	Infant	5.34 (1.28, 22.27)	3.56 (1.41, 8.95)	2.79 (0.35, 21.81)
	Neonate	2.36 (0.48, 11.50)	2.83 (1.05, 7.65)	2.02 (0.21, 19.62)
	Toddler	2.80 (0.63, 12.45)	2.24 (0.85, 5.89)	3.60 (0.45, 28.64)
	Pre-school	5.56 (1.21, 25.43)	2.42 (0.83, 7.02)	4.04 (0.44, 36.52)
	School-age	1.87 (0.40, 8.76)	1.41 (0.515, 3.89)	2.5 (0.29, 20.91)
	Older	Ref		
Source of admission	ED admission	0.56 (0.36, 0.87)	0.68 (0.49, 0.95)	0.68 (0.34, 1.35)
	Intra-hospital	Ref		
Date of admission	January-March	1.36 (0.74, 2.47)	0.45 (0.29, 0.70)	1.19 (0.46, 3.05)
	April-June	1.27 (0.68, 2.34)	0.37 (0.23, 0.60)	1.13 (0.43, 2.95)
	July-September	0.98 (0.49, 1.96)	0.54 (0.34, 0.85)	1.09 (0.39, 3.04)
	October-December	Ref		
Diagnosis	ICD-10 (I): Certain infectious and parasitic diseases	Ref		
	ICD-10 (II): Neoplasms	8.63 (0.82, 90.53)	2.21 (0.22, 21.83)	28.80 (2.28, 362.6)
	ICD-10 (III): Diseases of the blood and blood-forming organs and certain disorders involving the immune mechanism	0.57 (0.17, 1.87)	0.22 (0.09, 0.53)	1.92 (0.42, 8.72)
	ICD-10 (IV): Endocrine, nutritional, and metabolic diseases	0.12 (0.027, 0.57)	0.12 (0.05, 0.277)	-
	ICD-10 (VI): Diseases of the nervous system	2.24 (1.02, 4.93)	0.54 (0.30, 0.98)	1.57 (0.34, 7.12)
	ICD-10 (IX): Diseases of the circulatory system	1.50 (0.63, 3.56)	1.06 (0.64, 1.75)	1.25 (0.25, 6.29)
	ICD-10 (X): Diseases of the respiratory system	1.13 (0.53, 2.43)	0.19 (0.10, 0.36)	0.68 (0.15, 3.10)
	ICD-10 (XI): Diseases of the digestive system	0.37 (0.04, 2.98)	0.38 (0.13, 1.11)	7.50 (1.83, 30.78)
	ICD-10 (XII): Diseases of the skin and subcutaneous tissue	8.63 (0.82, 90.49)	-	-
	ICD-10 (XIV): Diseases of the genitourinary system	1.72 (0.36, 8.25)	0.22 (0.02, 1.66)	8.633 (1.66, 44.7)
	ICD-10 (XVI): Certain conditions originating in the perinatal period	0.26 (0.03, 2.07)	0.067 (0.009, 0.49)	-
	ICD-10 (XVII): Congenital malformations, deformations, and chromosomal abnormalities	2.15 (0.255, 18.26)	2.21 (0.67, 7.2)	-
	ICD-10 (XVIII): Symptoms, signs, and abnormal clinical and laboratory findings, not classified elsewhere	-	0.53 (0.15, 1.83)	2.33 (0.23, 23.02)
	ICD-10 (XIX): Injury, poisoning, and certain other consequences of external causes	1.39 (0.53, 3.60)	0.71 (0.38, 1.32)	2.89 (0.68, 12.29)
	ICD-10 (XX): External causes of morbidity and mortality	1.52 (0.18, 12.60)	1.17 (0.32, 4.18)	-

## Discussion

Based on our findings, the source of admission plays a key role in influencing the health outcomes of patients who are admitted to the ICUs. Our analysis showed a link between the source of admission and mortality rates experienced in PICUs. This relationship is not co-dependent. The results showed that several other factors tended to influence the mortality rates as well, especially the type of diagnosis, age group, and even the period of the year that the patients are admitted. The mortality rate (average 6%) among the total health outcomes recorded and analyzed was not significant compared to the number of patients discharged (89%). Our findings showed that 81 (53.3%) deaths were from ED admissions while 71 (46.7%) deaths were from intra-hospital transfers, which shows that ED admission-related mortality cases are more frequent when compared to intra-hospital transfers (but lower as a percentage of total ED admissions). These results are consistent with the findings of previously published literature.

Odetola et al. [8] conducted an observational study to analyze the correlation between the source of admission and mortality among those admitted to PICUs. They revealed that the patients who were directly admitted to ICUs had lower odds of mortality compared to those admitted from the wards. The patients transferred from wards had higher odds of mortality when compared to those directly admitted to ICUs. Their study proposed better strategies to decrease the risk of mortality among those admitted from the wards. A previous investigation by Knaus et al. [17] also described the phenomenon of variance in admission outcomes as influenced by different admission sources. Hill et al. [7] also elaborated on the above-discussed concept of determining outcomes based on admission sources. However, Kudlow et al. [18] did not agree with earlier researchers' findings in terms of associating mortality with the source of admission. Their study focused on inter-hospital transfers and found no positive evidence linking mortality risk with admission sources. Golestanina et al. [12] endorsed Kudlow's findings and did not support the concept of associating mortality risk with admission sources. Nevertheless, they concluded that the proper utilization of sources and differences in the duration of hospital stay were significant factors in determining the relationship between mortality and outcomes related to sources.

Gregory et al. [19] demonstrated that children admitted from other hospitals needed intensive care more than children transferred from the same hospital. However, there was no clear difference in mortality rates between the two groups. In another study, Odetola et al. [14] focused on the type and cause of admission to determine the outcomes related to sources. They observed that trauma admission after an injury might have a greater mortality rate compared to inter-hospital transfers. 

This study has some limitations. Primarily, it was retrospective in nature, which may have aggravated the risk of bias. Secondly, we included our patients as a convenience sample without randomization.

## Conclusions

Our findings showed that sources of admission, clinical condition, and, to a lesser extent, the age of the patients and date of admission might be used as independent predictors for determining the outcomes of admission, including discharge, long-stay, and mortality rates. Further studies are required to confirm these findings.
